# Surgery

**Published:** 1859-03

**Authors:** 


### SURGERY.

### XII.—*On Two New Methods of Treating Diseases of the Lachrymal Sac.* By Dr. v. Grafe.

In one of the sessions of the *Society of Berlin Physicians*, (July,) Dr. v. Gräfe reported on two new methods of treating affections of the lachrymal sac, which he considers a decided progress in ophthalmic surgery. One of them was proposed by Bowman, and has for its object the restoration of the permeability of the lachrymal passages by methodic dilatation. It differs from all the known methods of dilatation in the point that the lachrymal sac is not laid open through the skin, but that the instruments are introduced from the mucous membrane through the inferior punctum lachrymale, which has been previously dilated by slitting it. Although an experience of only four months does not permit any positive statement on the permanency of cures thus obtained, Dr. v. Gräfe does not hesitate, even at this early moment, to pronounce Bowman's method the best of all used for the restoration of the lachrymal passages.

The second innovation was proposed by Dr. Tavignot, and has the opposite indication in view, viz., to destroy the lachrymal passages. Believing that the entrance of tears rendered the obliteration of the lachrymal sac difficult, Tavignot recommends to cut off the puncta lachrymalia, in order to prevent tears passing into the sac. The idea itself is correct, but the obliteration of the lachrymal canals is not effected with certainty by the process recommended. Von Gräfe uses other means, for instance ligation with a suture, gradually cutting through, or cauterization by means of a small *portes caustiques*, which are introduced into the lachrymal canals. Dr. Leibreich, who assisted in Gräfe's clinic, conceived the idea of coating Anel's probes with nitrate of silver; in order to make the caustic adhere, the probes were first rendered rough by exposing them to the action of nitric acid; thus prepared, they were dipped into fused nitrate of silver. Any silver instrument can be converted by this process into a caustic body. After permeability of the lachrymal canals is obtained, the obliteration of the lachrymal sac is easily effected by gentle caustics. The hot iron, chloride of zinc, Vienna paste, etc., which often produce circumscribed caries, can be dispensed with.

By these two innovations the old contest between destructive and conservative surgery, in the treatment of diseases of the lachrymal sac, has been revived. According to Dr. v. Gräfe's opinion, the following rules are to be observed in regard to the indications:—1. In every case in which circumstances offer the prospect that perviousness may be permanently restored, the surgeon should endeavor to obtain it by Bowman's method. 2. In cases where the restoration of permeability is problematic, and could only be obtained by a tedious cure, it must be ascertained whether the lachrymal glands of the patient, after removal of all causes stimulating them to excessive secretion, furnish a relatively great or small quantity of tears. Dr. v. Gräfe gives the necessary rules for making this estimate. In cases in which the quantity of the secretion is small, obliteration, after cauterizing the lachrymal canals, is preferable to restoration of the continuity. No stillicidium lachrymarum remains in this case. If, however, the quantity of the secretion is large, Bowman's method should be first tried, for fear that the stillicidium might remain; only if it is impossible to obtain a permanent cure by this means, the lachrymal sac should be obliterated. Dr. v. Gräfe communicates the following statistical results in reference to this operation:—Of one hundred patients in whom the lachrymal sac has been successfully destroyed, twenty suffer of permanent and troublesome overflowing of tears; seventy are molested neither at their work nor in the room, but experience increased moistening in open air, or if excited to tears, etc.; ten finally do not notice any difference from the normal eye. 3. In cases of caries, organic obstructions, etc., in which there is no prospect for restoration of the continuity, the lachrymal sac should be at once obliterated, as in any case the condition of the patient is ameliorated by this measure. Thus the troublesome suppuration is not only done away with, but some of the principal causes of the hypersecretion of tears are also removed, and in consequence of it the stillicidium is proportionately reduced.—(*Allgemeine Medezin. Central Zeitung*, 1858, 67.)

XIII.—*On the Use of Ergot in Certain Diseases of the Eye.* By DR. V. WILLEBRAND.

The known effect of ergot upon the unstriped muscular fibres of the uterus, and upon the analogous tissues of the walls of the blood-vessels, induced Dr. v.

Willebrand to try this remedy in several forms of diseases of the eye, in which he supposed that the evil could be cured by exciting a greater contractility in the walls of the blood-vessels, or in other tissues containing unstriped muscular fibres. The greatest benefit from the administration of ergot, the author derived in those disturbances of the function of accommodation, described as *hebetudo visus*, (asthenopia,) in which the eye can accommodate itself only for a short time sufficiently to occupations near to the face, (reading, writing, sewing, etc.) Dr. v. Willebrand met serious cases of this kind, particularly among the pupils of young ladies' seminaries, who are generally exposed to unfavorable conditions of ocular hygiene, such as writing with the body bent forward, and with insufficient light. The author cured all these cases, at least for some time, by the exhibition of ergot; at the same time he ordered the eyes to be spared, and spectacles dampening the light to be used. The author used the remedy successfully also in a case of epophthalmus with hypertrophy of the heart and of the thyroid gland, (a peculiar combination which has been lately much discussed,) and in blepharitis, and pustular syndermitis of children. As the usual dose for adults, he prescribes five grains of ergot, generally combined with magnes. carbon., sometimes, if chlorosis is present, with iron, to be given three or four times daily.—(*Archives für Ophthalmologie*, 1858, iv.)

### XIV.—*Observations on the Causes, Nature, and Treatment of Hemeralopia.* By DR. NETTER.

Dr. Netter, Physician to the Military Hospital of Strasburg, had the opportunity of observing a great many cases of hemeralopia in the French army, and presented his experiences to the *Académie des Sciences*, in an interesting memoir on this disease. The conclusions to which he arrives are the following:—1. Hemeralopia is that kind of blindness opposite to nyctalopia, and is caused by an excess of light, while the latter originates from a long privation of this stimulus. 2. When a patient afflicted with hemeralopia is conducted into a very dark room during daylight, he is unable to see, although the persons accompanying him recognize every object in the room. Hemeralopia is, therefore, not a periodical blindness, which commences in the evening and disappears in the morning, but exists also during daytime, and consists in the incapability of seeing by diminished light; in short, it is a blindness in the dark without the least reference to the time of the day. 3. The cure of the disease can be accomplished in a few hours. The patients are brought into a dark room in the middle of the day, and are ordered to look about continually in all directions, so as to exert their power of vision. After two or three hours, the faculty of seeing returns: with this result the disease disappears entirely, and no relapse takes place, not even during the following nights.—(*Comptes Rendus*, 1858.)

### XV.—*On the Internal Treatment of Ulcers of the Leg by Iodide of Potassium.* By M. TRASTOUR.

Without reviewing the theory of an "ulcerative diathesis," advanced by Phil. Boyer, M. Trastour is of the opinion that ulcers of the leg are owing as often to a general disposition of the economy, as to local causes, and this opinion seems to him justified by the success which he has obtained from iodide of potassium in the treatment of these ulcers. Repeated observations permit him to draw up the following propositions :—1. Iodide of potassium, administered in a dose of two to six grammes a day, cures the most obstinate ulcers of the leg in one or two months, rarely more, no matter whether they are of syphilitic nature or not. The ulcers, and even the varicose congestion, yield rapidly to this medication, which may be assisted by regulated compression and a simple dressing. 2. The patients can continue their occupation during the treatment; they do not require rest. 3. The cure by this simple method is easier, more complete and reliable, than by any other known method.

There are, however, a certain kind of ulcers which require a special and more complex treatment; these are the scorbutic, herpetic, and scrofulous ulcers. Of ulcers which did not present one of these peculiar characters, M. Trastour reports several cases which speak highly in favor of his mode of treatment. The modification which takes place in the ulcers by the use of iodide of potassium may perhaps be explained by supposing that this salt, which passes rapidly into all the secretions, passes also into the ulcerous secretion. Brought in contact with the ulcers from within to without, it modifies them, molecule after molecule, that is to say, as completely as possible; at the same time, it acts upon the surrounding congestion, and upon the whole constitution.—(*Journal de la Société Académique de la Loire-Inférieure*, tome 34, l. 177, and *Gaz. Hebdomad.*, December, 1858.)

XVI.—*Modification in the Preparation of Moxas.* By Dr. Cramer.

After having made moxas from carded cotton, in the usual way, Dr. Cramer applies to both ends of them a layer of collodion by means of a brush, and allows them to dry. Thus prepared, they preserve their density during the process of combustion, and do not dilate even toward the end, as ordinary moxas do; their combustion is neither too rapid nor too slow. In applying them, one end is ignited, and the other is glued to the skin by means of one or two drops of collodion; the combustion is hastened by one of the usual measures. This modification has given very favorable results in the hands of Dr. Cramer, and recommends itself from its simplicity.—(*Echo Médical Suisse*, 1858, tome 1, p. 762.)

